# Involvement of oxidative stress in tri-ortho–cresyl phosphate-induced autophagy of mouse Leydig TM3 cells *in vitro*

**DOI:** 10.1186/s12958-016-0165-x

**Published:** 2016-06-07

**Authors:** Xiaomei Liu, Linlin Xu, Jingcao Shen, Jinglei Wang, Wenli Ruan, Mei Yu, Jiaxiang Chen

**Affiliations:** Department of Physiology, Medical College of Nanchang University, 461 Bayi Road, Donghu District, Nanchang, 330006 People’s Republic of China; Medical Research Center, The First Affiliated Hospital of Nanchang University, Nanchang, 330006 People’s Republic of China; Library, Medical College of Nanchang University, Nanchang, 330006 People’s Republic of China

**Keywords:** Tri-ortho–cresyl phosphate, Leydig cells, Autophagy, Oxidative stress

## Abstract

**Background:**

As a plasticizer, plastic softener, and flame-retardant, tri-ortho–cresyl phosphate (TOCP) is and has been widely used in industry and reported to have a toxic effect on the male reproductive system in animals besides neurotoxicity and immunotoxicity. We have reported that TOCP inhibits spermatogenesis and induces autophagy of rat spermatogonial stem cells, but it is still unknown whether TOCP induces autophagy of mouse Leydig cells and its potential mechanism.

**Methods:**

Cell viability was observed by MTT assay. Level of testosterone was measured by radioimmunoassay. Apoptosis was observed by AnnexinV-FITC/PI assay. The contents of LC3, Atg5-Atg12, and Beclin 1 were detected by Western blotting analysis. Autophagosomes were investigated by transmission electron microscopy. The contents of MDA and GSH and the activities of SOD, GSH-PX, total antioxidant status (TAS) and total oxidant status (TOS) were measured by oxidative stress kits.

**Results:**

The present study shows that TOCP markedly inhibited viability and testosterone output of mouse Leydig TM3 cells but had no effect on apoptosis. However, TOCP significantly increased both LC3-II and the ratio of LC3-II to LC3-I and the contents of autophagy proteins Atg5 and Beclin 1. Transmission electron microscopy (TEM) showed that TOCP increased autophagic vacuoles of the cytoplasm, indicating that TOCP could induce autophagy of the cells. TOCP significantly induced oxidative stress of mouse Leydig TM3 cells. H_2_O_2_ also inhibited viability and induced autophagy of the cells; however, inhibition of oxidative stress by N-acetyl-L-cysteine (NAC) could rescue the inhibition of cell viability and induction of autophagy by TOCP.

**Conclusions:**

The results show oxidative stress might be involved in TOCP-induced autophagy of mouse Leydig TM3 cells.

## Background

Tricresyl phosphate (TCP) has been widely used as plastic softeners, plasticizers, jet oil additives, and flame retardants in industry, and tri-ortho–cresyl phosphate (TOCP) is the important one of three isomers (i.e., o-, m-, or p-cresyl) [1, 2]. It has been shown that TOCP mainly induces a delayed neurodegenerative condition known as OP-induced delayed neuropathy (OPIDN). OPIDN is characterized by sensory impairment, ataxia, weakness, muscle fasciculation, hyporeflexia, and even progressive spastic paraplegia by affecting both the central and peripheral nerves in sensitive species [3, 4]. TOCP reportedly can inhibit viability of SH-SY5Y cells [5, 6] and induces autophagy of the cell [7].

TOCP has been shown to induce reproductive toxicology [8, 9] besides neurotoxicity [1, 10], immunotoxicity [11, 12], and liver toxicity [13]. It has been shown to disrupt the seminiferous epithelium in rats [8, 9] and decrease sperm motility and sperm number in both roosters [14] and rats [9, 15]. TOCP can also lead to a decrease in the fertility index and the number of liveborn pups per litter in Swiss (CD-1) mice [16]. We found that TOCP disrupts the seminiferous epithelium in the testis, decreases sperm density of the epididymis in mice [17], and induces autophagy of rat spermatogonial stem cells [18].

Spermatogenesis is a complex process generating functional sperm in the testis, which consist of sequential and highly organized steps of undifferentiated spermatogonial stem cell proliferation and differentiation [17–19]. Leydig cells play an important role in maintaining spermatogenesis besides Sertoli cells and can be affected by many chemicals [20, 21]. The toxicity of TOCP in vivo mainly results from its metabolite saligenin cyclic-o-tolyl phosphate (SCOTP), which is converted by cytochrome P450 [22]. SCOTP can inhibit viability of mouse spermatogonial stem cells [17] and induce autophagy of rat spermatogonial stem cells [23]. It shows that Leydig cells highly express functional CYP450 in mature rat testes [24], which indicates that TOCP might cause toxic effects in Leydig cells. Chapin et al found that testosterone output was decreased after primary rat Leydig cells were treated with TOCP, which was replicated by subsequent in vivo experiments [25]. It shows that oxidative stress can be induced by TOCP in the cerebrum, spinal cord, and sciatic nerve of hens and male mouse liver [10, 13]. However, it remains unclear what the actual effect and mechanism of TOCP is on Leydig cells, including its potential mechanism.

The aim of the present study is to investigate whether oxidative stress is involved in TOCP-induced autophagy of mouse Leydig TM3 cells. This study sets in motion our future investigation of the mechanisms underlying TOCP inhibition of spermatogenesis.

## Methods

### Reagents

TOCP (purity > 99.0 %) was purchased from BDH Chemicals Co. Ltd (Poole, England). Mouse Leydig TM3 cells were purchased from the Cell Culture Center of the Institute of Basic Medical Science, Chinese Academy of Medical Sciences (Beijing, China). Cell culture reagents were obtained from Gibco BRL (Grand Island, NY, USA). An AnnexinV-FITC Apoptosis Detection Kit was obtained from Invitrogen Life Technologies (Oregon, USA). Rabbit anti-LC3 polyclonal antibody (PD014), rabbit anti-Atg5 polyclonal antibody (PM050), and rabbit anti-Beclin 1 polyclonal antibody (PD017) were obtained from MBL Co. Ltd (Nagoya, Japan). Mouse anti-β-actin monoclonal antibody, goat anti-mouse IgG-HRP, and goat anti-rabbit IgG-HRP were purchased from Santa Cruz Biotechnology (Santa Cruz, CA, USA). The enhanced chemiluminescence (ECL) reagent was obtained from Pierce Biotechnology (Rockford, IL, USA). Commercial oxidation-antioxidation assay kits of malondialdehyde (MDA), glutathione (GSH), superoxide dismutase (SOD), glutathione peroxidase (GSH-PX), total antioxidant status (TAS), and total oxidant status (TOS) were bought from Nanjing Jiancheng Bioengineering Institute (Nanjing, China). N-acetyl-L-cysteine (NAC) was purchased from Sigma (St. Louis, MO, USA).

### Cell culture

Mouse Leydig TM3 cells were grown and maintained in Dulbecco’s modified Eagle’s medium (DMEM) supplemented with 10 % fetal bovine serum, 100 IU/ml penicillin, and 100 μg/ml streptomycin. Incubations were carried out at 37 °C in a humidified atmosphere of 5 % CO_2_/95 % air. The cells were maintained in the logarithmic phase of growth and sub-cultured at 3–4-day intervals.

### MTT reduction assay

The cells (1 × 10^4^ cells/well) were seeded in a 96-well culture plate and incubated with fresh medium containing 0–200 μM H_2_O_2_ or 0–0.5 mM TOCP in the presence or absence of 5 mM N-acetyl-L-cysteine (NAC) for 48 h. TOCP was dissolved in dimethyl sulfoxide (DMSO); the final concentration of DMSO in the culture medium was 0.1 % (v/v). Forty-eight hours later, cell viability was assessed by MTT assay. Cell medium containing 0.5 mg/mL MTT was replaced in each well and incubated at 37 °C in 5 % CO_2_/95 % air for 4 h. The formazan formed was dissolved in DMSO, and the absorbance was measured in a spectrophotometer at 570 nm with a background reading of 660 nm.

### Detection of testosterone content by radioimmunoassay (RIA)

The concentration of testosterone in Leydig cells was determined using an RIA kit. The cell suspensions were incubated with 0–0.5 mM TOCP in the presence of hCG (0.05 IU/ml) at 37 °C for 48 h. The testosterone concentrations in culture medium were determined by an RIA kit according to the manufacturer’s instructions (Nanjing Jiancheng Bioengineering Institute, Nanjing, China).

### AnnexinV-FITC/PI apoptosis assay

The apoptosis assay was analyzed by double staining the cells with FITC-labeled AnnexinV and propidium iodide (PI), using an AnnexinV-FITC Apoptosis Detection Kit, according to the manufacturer’s instructions. In brief, mouse Leydig TM3 cells treated with 0–0.5 mM TOCP were collected and washed twice with phosphate-buffered saline (PBS). Then the cells were resuspended with the AnnexinV binding buffer and transferred to test tubes containing FITC-labeled AnnexinV and PI. The cells were then incubated in the dark for 15 min at room temperature and analyzed by flow cytometry, using the FACS Calibur system (BD Biosciences, San Jose, CA, USA). The excitation wavelength was 488 nm, and the emission wavelength was 530 nm. A total of 10,000 cells were acquired. Flow cytometric data were analyzed using FlowJo 7.6 software and displayed in dot plot of AnnexinV/FITC (*y*-axis) against PI (*x*-axis). The normal healthy cells were AnnexinV/FITC and PI double-negative, whereas the late apoptotic or secondary necrotic cells were double-positive. The early apoptotic cells were only AnnexinV/FITC positive, whereas the isolated nuclei or cellular debris were only PI positive [26].

### Western blotting analysis

The cells were trypsinized, washed twice with ice-cold PBS, and harvested in cell lysis buffer (50 mM Tris pH 7.5, 0.3 M NaCl, 5 mM EGTA, 1 mM EDTA, 0.5 % Triton X-100, 0.5 % NP40) containing protease inhibitor cocktail (Huatesheng Biotech, Fushun, Liaoning, China). Cell lysates were briefly ultra-sonicated and clarified by centrifugation at 12,000 × g for 10 min at 4 °C. The supernatants were collected for further experiments. Protein concentration was determined according to the method of Lowry et al. using bovine serum albumin (BSA) as a standard [27]. The protein samples were separated by sodium dodecyl sulfate-polyacrylamide gel electrophoresis with a 5 % stacking gel and 8 % separating gel and transferred to polyvinylidene fluoride (PVDF) membrane (Millipore Corporate, Billerica, MA, USA). Following transfer, membranes were blocked with 1× Tris-buffered saline (TBS) buffer containing 0.05 % Tween 20 and 5 % nonfat milk for at least 1 h at room temperature, then incubated with primary antibodies (diluted 1:1000), and finally incubated with horseradish peroxidase-conjugated goat anti-rabbit IgG (diluted 1:5000). Immunoreactive bands were detected using a ChemiDoc XRS system (Bio-Rad, Hercules, CA, USA) and standard ECL reagents.

### Transmission electron microscopy analyses

The cells were incubated in normal DMEM medium or treated with TOCP at 1.0 mM for 48 h or were starved for 2 h at 37 °C in a humidified incubator with 5 % CO_2_ in a starvation media (140 mM NaCl, 1 mM CaCl2, 1 mM MgCl2, 5 mM glucose, and 20 mM HEPES at pH 7.4 supplemented with 1 % (*w*/*v*) fresh BSA). At the end of incubation, the cell monolayers were washed with PBS and scraped gently with a plastic cell scraper. Then the harvested cells were pelleted by centrifugation at 800 rpm for 10 min and fixed in ice-cold 2.5 % glutaraldehyde for 2 h. Afterward, samples were post-fixed in 1 % OsO4 for 1 h, dehydrated through an ethanol series, and embedded in epoxy resin; then ultra-thin sections (60 nm) were double stained with uranyl acetate and lead citrate. Representative areas were cut and examined by transmission electron microscope (Hitachi H800).

### Measurement of oxidative stress in mouse Leydig TM3 cells

Mouse Leydig TM3 cells (1 × 10^6^ cells) were seeded in a 60 mm culture plate and incubated with fresh medium containing 0–0.5 mM TOCP for 48 h. The cells were homogenized and centrifuged at 600 g for 10 min at 4 °C, and then the supernatants were analyzed for the contents of MDA and GSH and the activities of SOD, GSH-PX, total antioxidant status (TAS) and total oxidant status (TOS), using kits according to the manufacturer’s instructions (Nanjing Jiancheng Bioengineering Institute, Nanjing, China). Protein concentration was assayed using the Bradford protein assay.

### Statistical analysis

Values are expressed as means ± SE. Data were evaluated by one-way analysis of variance (ANOVA) with a Newman–Keuls multiple range test. A difference between means was considered significant at a value of *P* < 0.05.

## Results

### TOCP inhibits cell viability of mouse Leydig TM3 cells

**Fig. 1:**
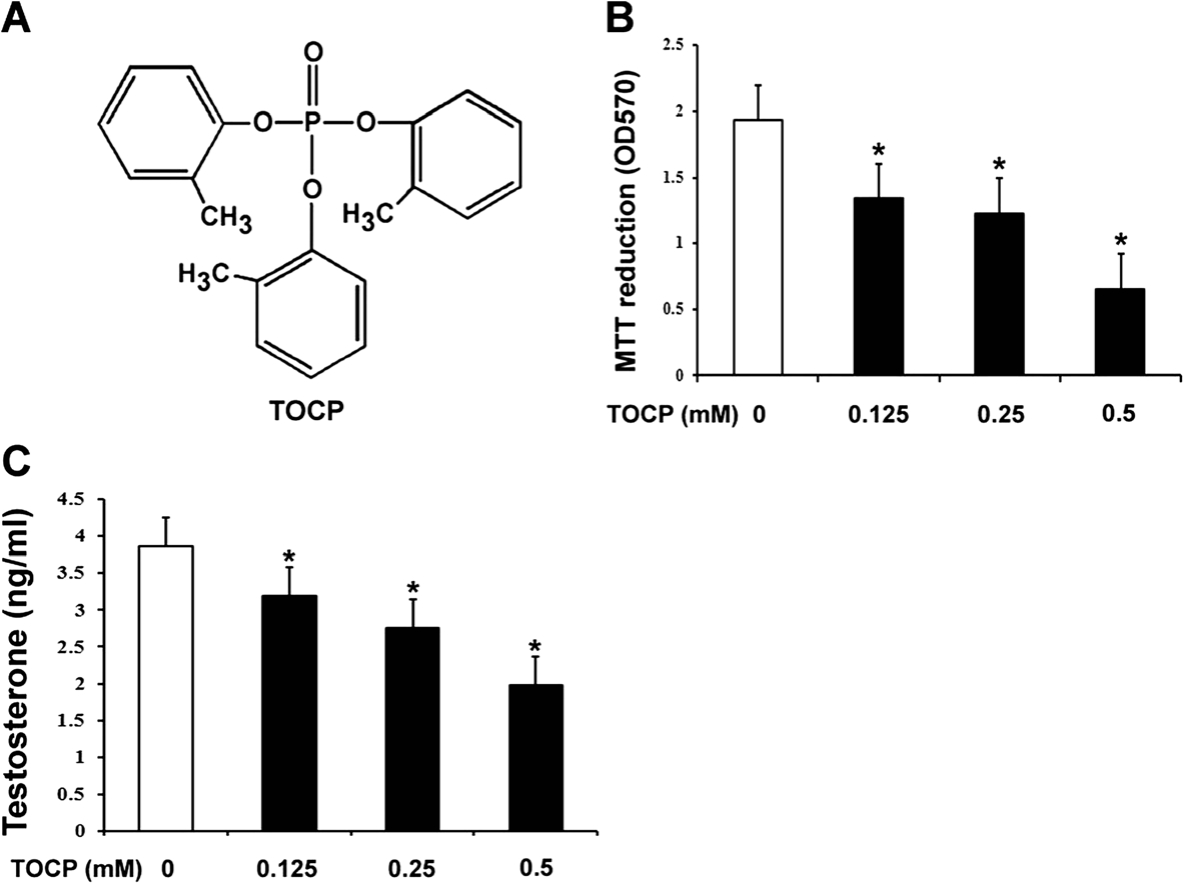
TOCP inhibits viability and testosterone output of mouse Leydig TM3 cells. **a** Chemical structure of tri-ortho–cresyl phosphate (TOCP). Mouse Leydig TM3 cells were treated with 0–0.5 mM TOCP for 48 h. Then, **b** cell viability was observed by MTT assay; **c** testosterone output was observed by RIA. The experiment was done in triplicate and repeated three times. Data were analyzed by one-way ANOVA. *P < 0.05

To observe whether TOCP inhibited viability of mouse Leydig TM3 cells, the cells were treated with 0, 0.125, 0.25, and 0.5 mM TOCP for 48 h. As shown in Fig. 1, TOCP inhibited cell viability of mouse Leydig TM3 cells in a dose-dependent manner. Furthermore, testosterone output was also inhibited by TOCP.

### Effect of TOCP on apoptosis in mouse Leydig TM3 cells

**Fig. 2:**
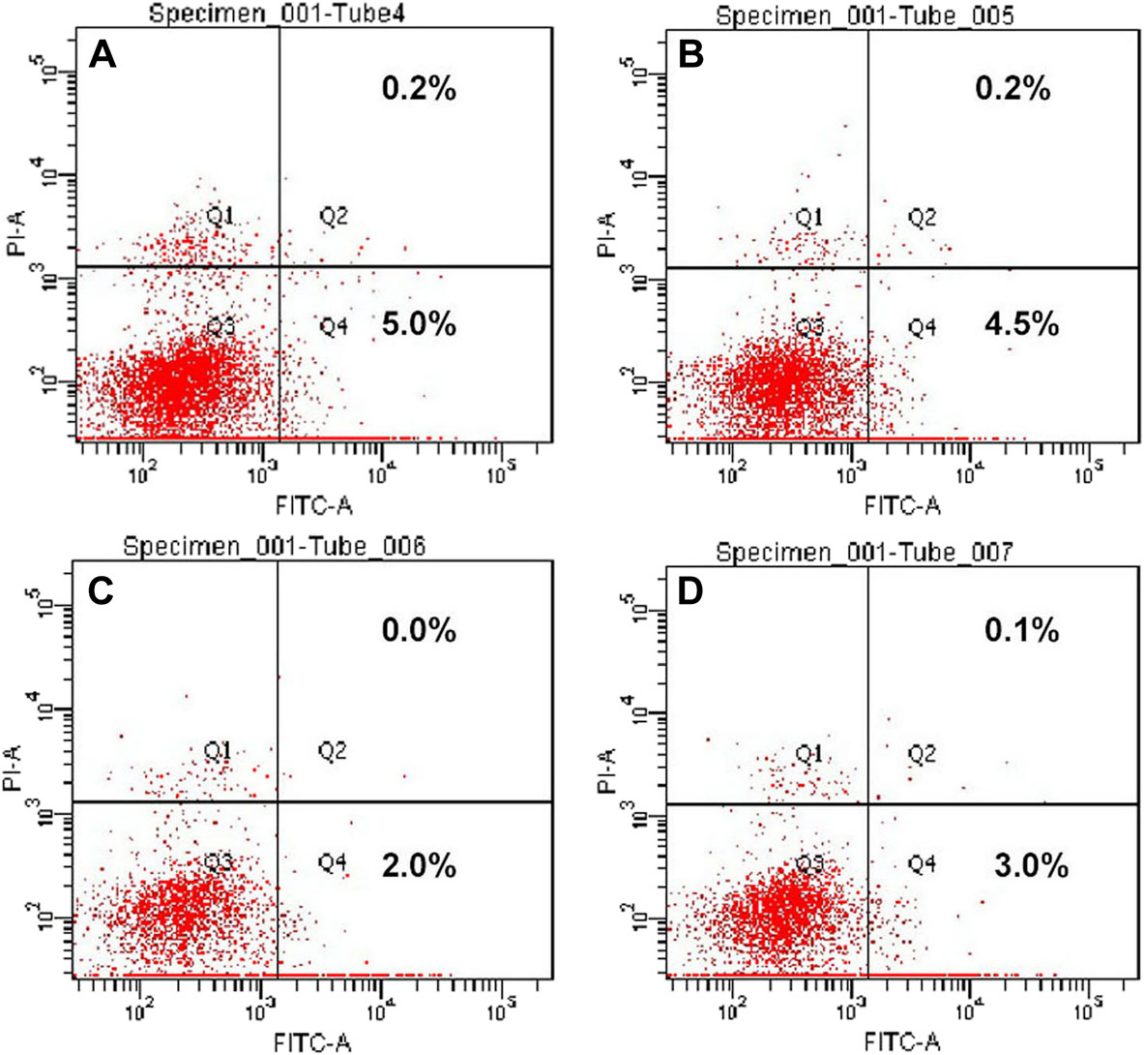
Effect of TOCP on apoptosis in mouse Leydig TM3 cells. Apoptosis was observed by flow cytometry method (FCM) assay after mouse Leydig TM3 cells were treated with 0 mM (**a**), 0.125 mM (**b**), 0.25 mM (**c**), or 0.5 mM (**d**) TOCP for 48 h

To confirm whether the anti-proliferative effect of TOCP resulted from induction of apoptosis, the number of AnnexinV-positive/PI-negative and AnnexinV-positive/PI-positive staining cells were counted by flow cytometry after the cells were treated with 0–0.5 mM TOCP for 48 h. As shown in Fig. 2, TOCP didn’t induce apoptosis of mouse Leydig TM3 cells. These results indicated that TOCP had no effect on apoptosis of mouse Leydig TM3 cells.

### TOCP induces autophagy in mouse Leydig TM3 cells

**Fig. 3:**
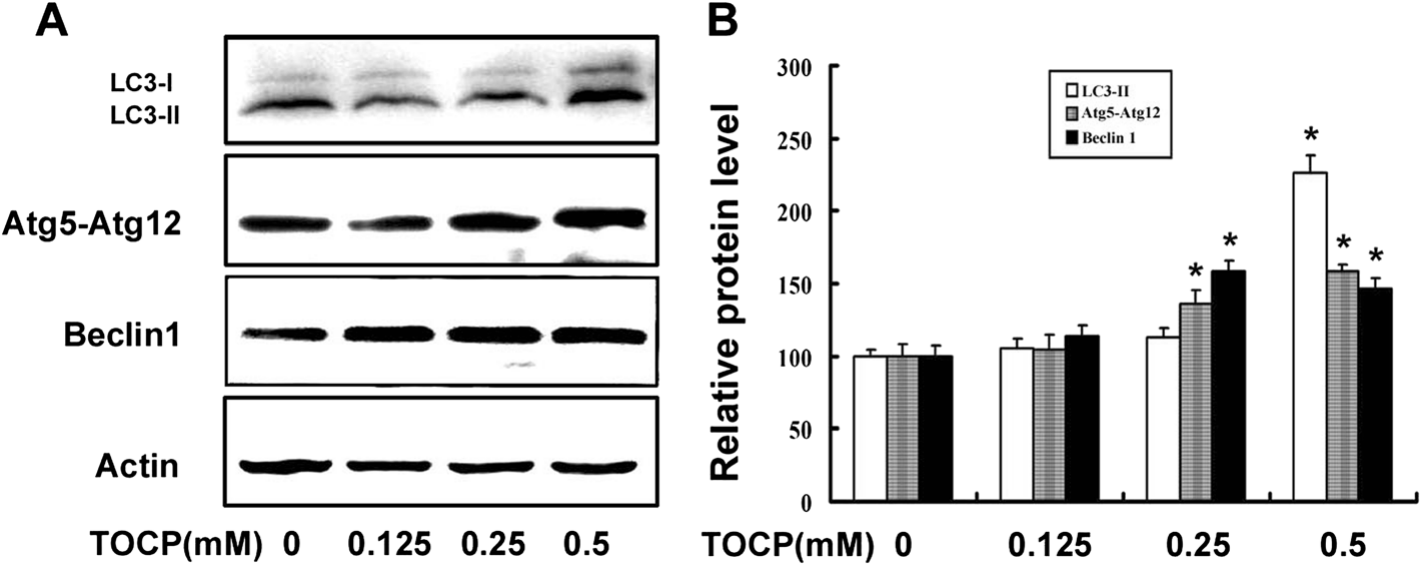
TOCP induces autophagy of mouse Leydig TM3 cells. Mouse Leydig TM3 cells were treated with 0–0.5 mM TOCP for 48 h. Then, the protein levels of LC3, Atg5–Atg12, and Beclin1 were observed by Western blot; actin was used as an internal control (**a**). The relative protein levels were quantified by densitometry and expressed as percentage of the control cells (**b**). The experiment was done in triplicate and repeated three times. Data were analyzed by one-way ANOVA. *P < 0.05

To evaluate whether TOCP induced autophagy in mouse Leydig TM3 cells, the autophagy-related proteins LC3, Atg5-Atg12, and Beclin 1 were analyzed by Western blot after the cells were treated with indicated concentration of TOCP for 48 h. As shown in Fig. 3, TOCP increased significantly both LC3-II and the ratio of LC3-II to LC3-I; the contents of Atg5–Atg12 and Beclin 1 were also increased in TOCP- treated cells.

**Fig. 4:**
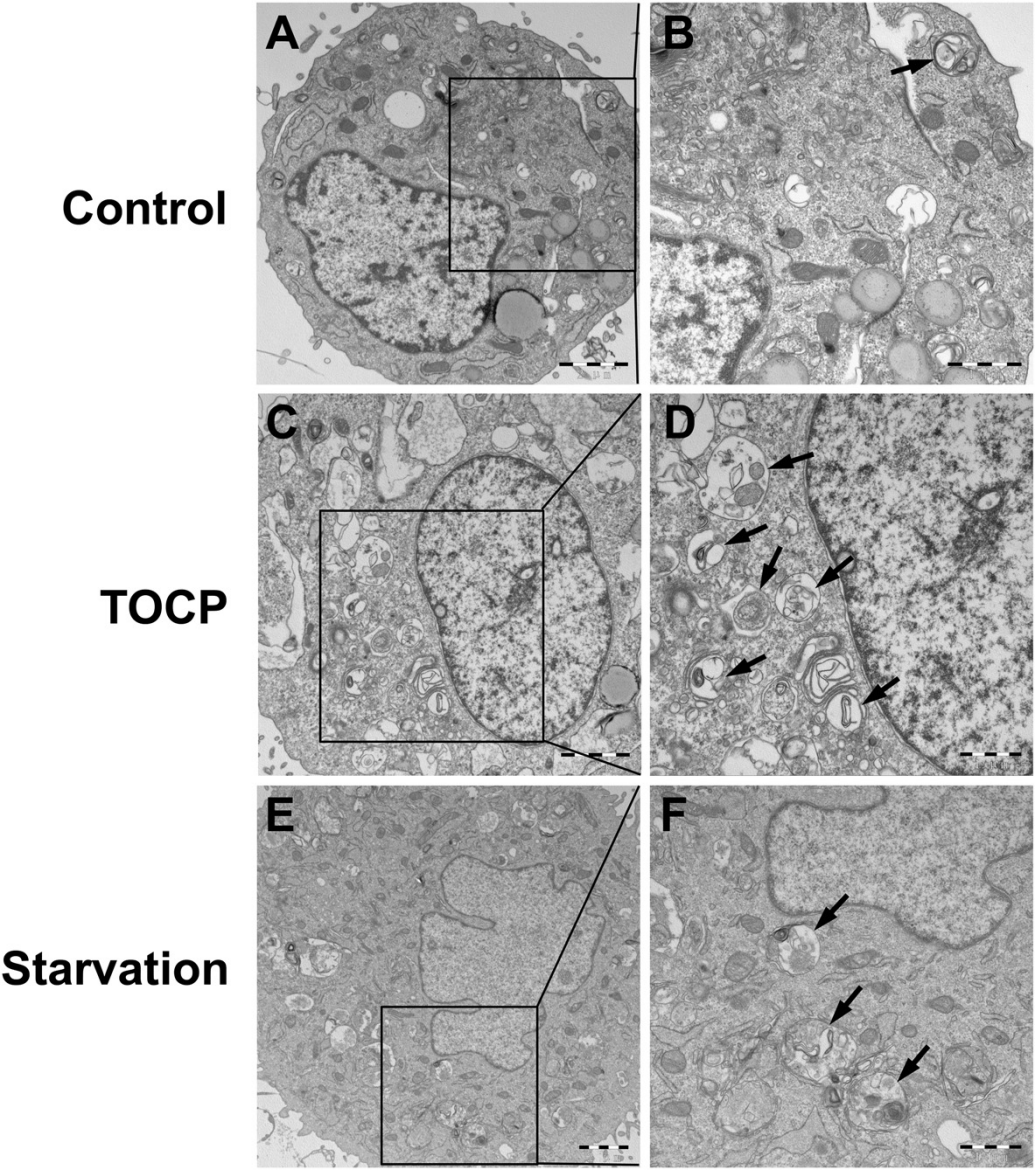
Ultrastructural evidence of TOCP inducing autophagy in mouse Leydig TM3 cells. Mouse Leydig TM3 cells were treated with DMSO (**a** and **b**) or 0.5 mM TOCP for 48 h (**c** and **d**). Then, autophagic vacuoles in the cells were visualized by transmission electron microscopy (TEM), starvation-treated cells as positive control (**e** and **f**). The autophagic vacuoles are indicated by arrows

The effect of TOCP on autophagy in mouse Leydig TM3 cells was further investigated by transmission electron microscopy (TEM). We found that there were relatively few autophagosomes in the cytoplasm of the control cells (Figs. 4a and b) but a significant increase in autophagic vacuoles in the cytoplasm containing extensively degraded organelles such as mitochondria and endoplasmic reticulum in TOCP-treated cells (Figs. 4c and d), and starvation (Figs. 4e and f). These results indicated that TOCP induced autophagy of mouse Leydig TM3 cells.

### TOCP induces oxidative stress in mouse Leydig TM3 cells

**Fig. 5:**
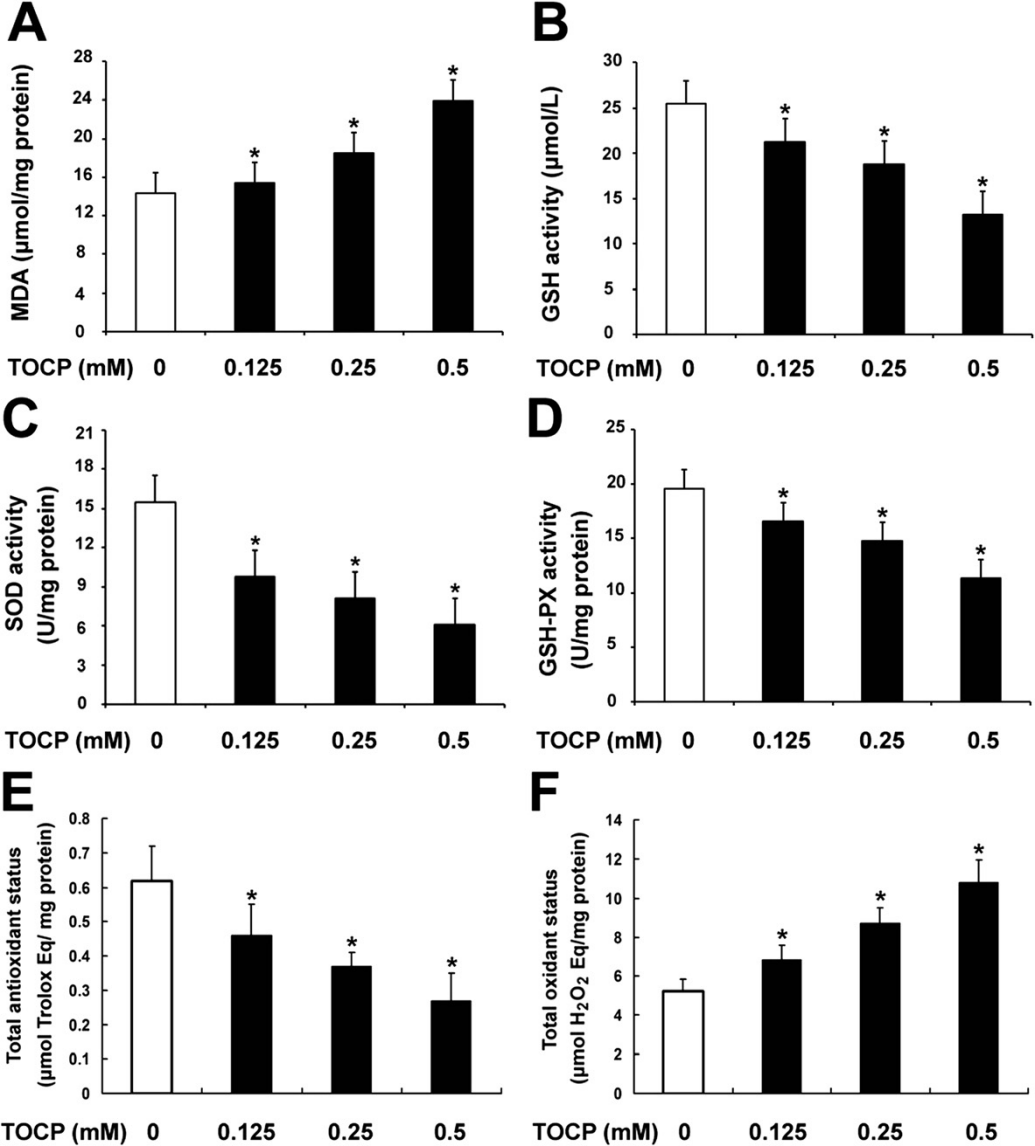
TOCP induces oxidative stress in mouse Leydig TM3 cells. Mouse Leydig TM3 cells were treated with 0–0.5 mM TOCP for 48 h; then the contents of MDA (**a**) and GSH (**b**), the activities of SOD (**c**) and GSH-PX (**d**), total antioxidant status (**e**), and total oxidant status (**f**) were determined. The experiment was done in triplicate and repeated three times. Data were analyzed by one-way ANOVA. *P < 0.05

To investigate whether oxidative stress was involved in TOCP-induced autophagy of mouse Leydig TM3 cells, the contents of MDA and GSH, the activities of SOD and GSH-PX, total antioxidant status (TAS), and total oxidant status (TOS) were determined after the cells were treated with 0–0.5 mM TOCP for 48 h. As shown in Fig. 5, TOCP significantly increased MDA and TOS level in the cells in a dose-dependent manner, whereas the content of GSH, activities of antioxidant enzymes SOD and GSH-PX, and TAS were dramatically decreased in the TOCP-treated cells, respectively. These results indicated that TOCP could induce oxidative stress in mouse Leydig TM3 cells.

### Involvement of oxidative stress in TOCP-induced autophagy of mouse Leydig TM3 cells

**Fig. 6:**
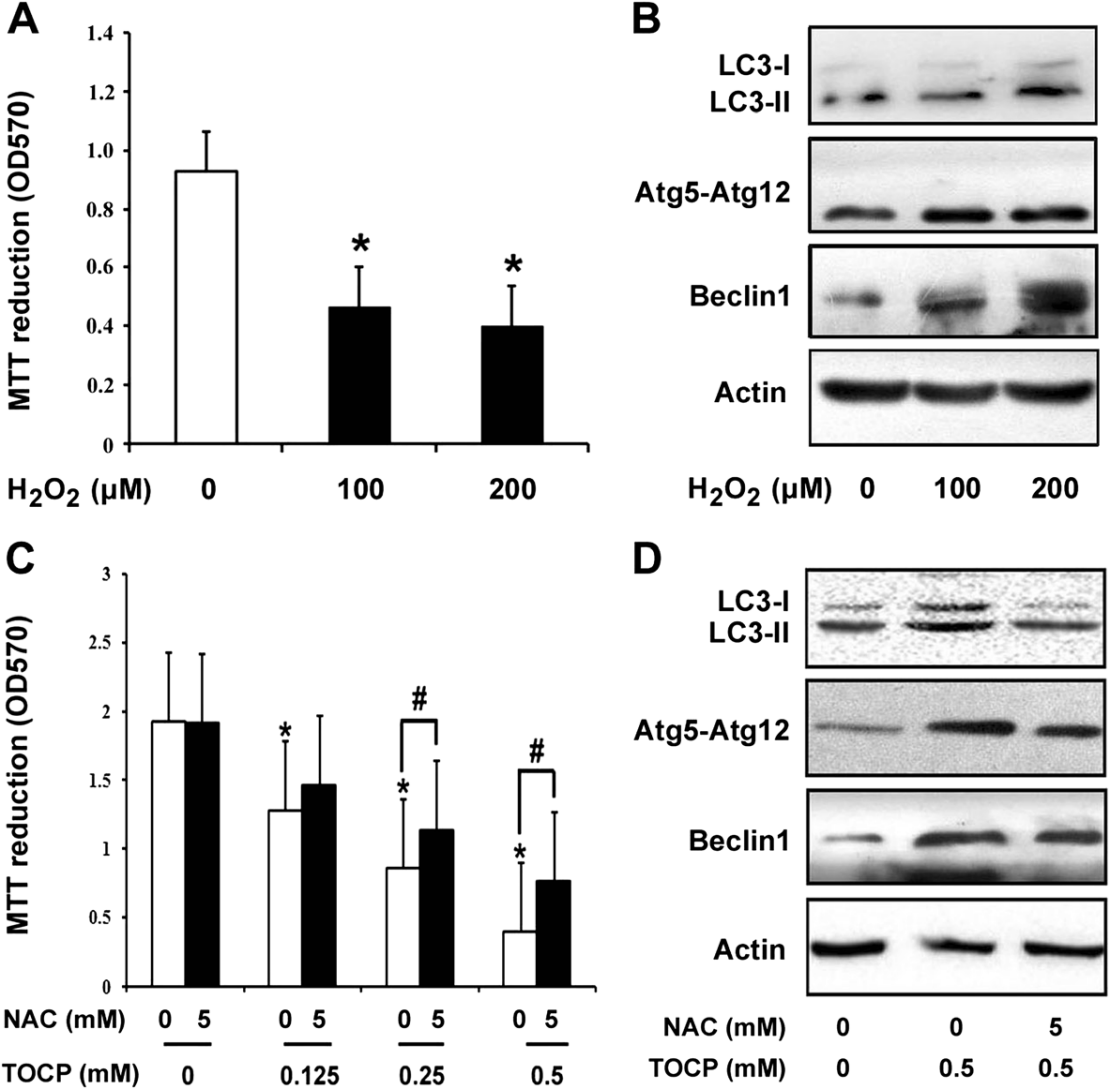
Oxidative stress is involved in TOCP-induced autophagy of mouse Leydig TM3 cells. Mouse Leydig TM3 cells were treated with 0–200 μM H_2_O_2_ for 48 h. Then, cell viability was observed by MTT assay (**a**), and the protein levels of LC3, Atg5–Atg12, and Beclin1 were observed by Western blot; actin was used as an internal control (**b**). The cells were treated with 0–0.5 mM TOCP for 48 h in the absence or presence of 5 mM NAC; then cell viability (**c**) and the contents of LC3, Atg5–Atg12, and Beclin1 (**d**) were observed by MTT assay and Western blot, respectively. The experiment was done in triplicate and repeated three times. Data were analyzed by one-way ANOVA. *P < 0.05 vs. 0 mM TOCP in the absence of NAC. #*P* < 0.05

**Fig. 7:**
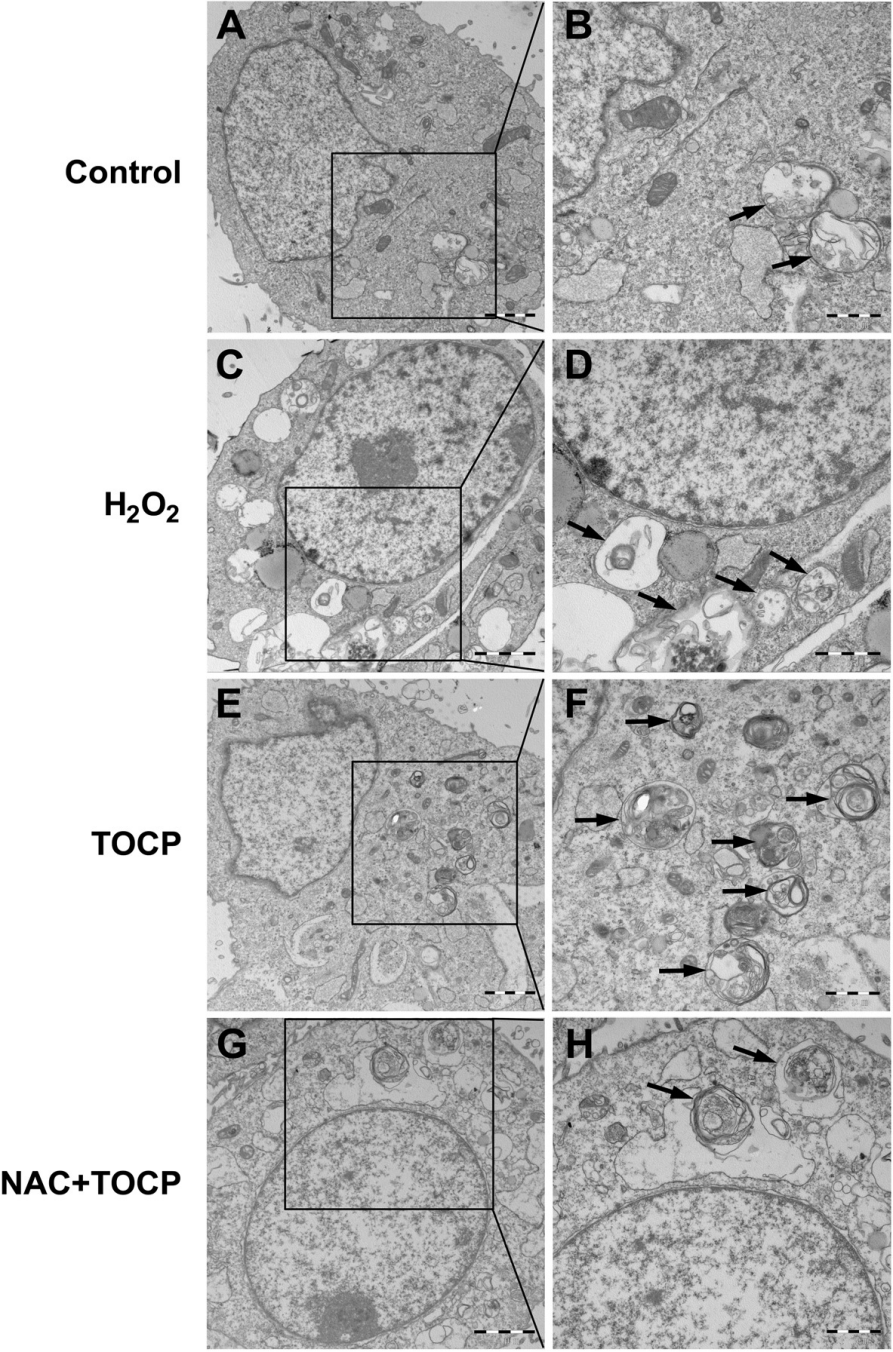
Ultrastructural evidence of involvement of oxidative stress in TOCP-induced autophagy of mouse Leydig TM3 cells. Mouse Leydig TM3 cells were treated with DMSO (**a** and **b**), 200 μM H_2_O_2_ (**c** and **d**), 0.5 mM TOCP for 48 h (**e** and **f**), or 5 mM N-acetyl-L-cysteine (NAC) plus 0.5 mM TOCP for 48 h (**g** and **h**). Then, autophagic vacuoles in the cells were visualized by transmission electron microscopy (TEM). The autophagic vacuoles are indicated by arrows

To determine further whether oxidative stress was involved in TOCP-induced autophagy of mouse Leydig TM3 cells, the cells were treated with 0, 100, and 200 μM H_2_O_2_ for 48 h. As shown in Fig. 6a, H_2_O_2_ markedly inhibited cell viability in a dose-dependent manner; and the contents of LC3-II, Atg5–Atg12, and Beclin 1 were significantly increased in H_2_O_2_-treated cells (Fig. 6b), indicating that oxidative stress could induce autophagy of mouse Leydig TM3 cells. Cell viability was also observed after the cells were treated with 0–0.5 mM TOCP for 48 h in the presence or absence of 5 mM N-acetyl-L-cysteine (NAC), an inhibitor of oxidative stress. It showed that TOCP inhibited viability of mouse Leydig TM3 cells, but inhibition of oxidative stress by NAC could rescue viability to a certain degree (Fig. 6c), implicating that oxidative stress was involved in TOCP inhibiting viability of mouse Leydig TM3 cells. Furthermore, the data also indicated that the inhibition of oxidative stress could also rescue autophagy as shown either by Western blot (Fig. 6d) or by TEM (Fig. 7). These results indicated that oxidative stress was involved in TOCP-induced autophagy of mouse Leydig TM3 cells.

## Discussion

In the present study, we provided evidence of the involvement of oxidative stress in TOCP-induced autophagy of mouse Leydig TM3 cells.

TOCP, a plastic softener, plasticizer, jet oil additive, and flame-retardant in industry, has been reported to have adverse effects on the male reproductive system besides neurotoxicity and immunotoxicity. We previously reported that TOCP decreases the sperm density in the epididymis and inhibits viability of spermatogonial stem cells in mice [17]. In the present study, we also found that TOCP could inhibit viability of mouse Leydig TM3 cells. The primary function of Leydig cells is to synthesize and secrete androgen, which is essential for male reproductive development and health [28]. Although many mechanisms are involved in synthesis and secretion of androgen, inhibition of the viability of Leydig cells might affect the output of testosterone. We have shown in this study that testosterone content of mouse Leydig TM3 cells was inhibited by TOCP in a dose-dependent manner, which is consistent with results of Chapin et al. in rat primary Leydig cells [25]. The inhibition of cell viability by TOCP might result from the induction of apoptosis. Many chemicals and plasticizers can induce apoptosis of spermatogenic cells and Leydig cells [29–31]. Interestingly, TOCP did not induce apoptosis of mouse Leydig TM3 cells.

Macroautophagy, hereafter referred to as autophagy, is a cellular degradative pathway that involves the delivery of cytoplasmic cargo to the lysosome [31, 32]. Autophagy occurs at low basal levels in cells to perform homeostatic functions such as protein and organelle turnover. It is rapidly upregulated when cells need to generate intracellular nutrients and energy during starvation, growth factor withdrawal, or high bioenergetic demands [33]. Under most circumstances, autophagy can promote cell survival during amino-acid deprivation or under stressful conditions such as neurodegenerative diseases, pathogen infections, and chemotherapy [34–36]. In certain cellular settings, however, autophagy is also considered a form of nonapoptotic programmed cell death called “type II” or “autophagic” cell death [36, 37]. Long et al. showed that TOCP induces autophagy of SH-SY5Y cells [38]. We also found that TOCP can induce autophagy of rat spermatogonial stem cells [18]. In this study, we found that TOCP could significantly increase both LC3-II and the ratio of LC3-II to LC3-I as well as Atg5–Atg12 and Beclin 1. TEM was then used to identify TOCP-induced autophagy further, which is the gold-standard method [39]. These results suggest that TOCP could induce autophagy of mouse Leydig TM3 cells.

Reactive oxygen species (ROS) are chemically reactive molecules containing oxygen, which generate as by-products of biological oxidations. ROS include both free radicals, such as nitric oxide (NO•), superoxide (O_2_•^−^), and hydroxyl radical (OH•), and peroxides [40]. ROS are generated during mitochondrial respiration under physiological conditions. ROS can be scavenged by reduced glutathione (GSH) and antioxidant enzymes such as superoxide dismutase (SOD) and glutathione peroxidase (GSH-PX). When there is an imbalance of ROS production and the cellular antioxidant defense system, oxidative stress occurs [41].

Oxidative stress has been implicated as a critical pathophysiological mechanism of reproductive toxicity from environmental chemicals or organophosphorus compounds (OPs) [42, 43]. TOCP is shown to induce oxidative stress in cerebrum, spinal cord, and sciatic nerves of hens [10]. Oxidative stress is also involved in TCOP-induced cytotoxicity in C6 glioma cells [44]. However, it was unclear whether TOCP might induce oxidative stress in mouse Leydig TM3 cells. To investigate such a hypothesis, assessment of malondialdehyde (MDA) and GSH, the activities of SOD and GSH-PX, total antioxidant status (TAS), and total oxidant status (TOS) were assessed after mouse Leydig TM3 cells were treated with TOCP. TOCP significantly increased MDA and TOS in the cells; there was a decrease in GSH and in the activities of SOD, GSH-PX, and TAS in the TOCP-treated cells. These results suggest that TOCP can induce oxidative stress in mouse Leydig TM3 cells.

Oxidative stress can induce autophagy and plays an important role in chemical- induced autophagy [45, 46]. In the current study, we found that H_2_O_2_ markedly inhibited cell viability and induced autophagy of mouse Leydig TM3 cells. However, inhibition of oxidative stress by NAC can rescue TOCP-induced autophagy and inhibition of cell viability to a certain degree. Collectively, these data provide evidence that oxidative stress may be important in TOCP-induced autophagy of mouse Leydig TM3 cells.

The mechanism of autophagy induced by chemicals is very complicated; many genes might be involved in chemicals- induced autophagy. TOCP might induce autophagy of mice Leydig cells via other signal transduction pathways besides ROS. Leydig cells highly express functional CYP450 and can convert TOCP to cyclic-o-tolyl phosphate (SCOTP) in mature rat testes, which will be more toxic to the cells [24, 25]. We also found that SCOTP can induce autophagy of rat spermatogonial stem cells [23]. However, it is unclear whether SCOTP can induce autophagy of mice Leydig cells. We will further study the mechanism of in TOCP- induced autophagy of mice Leydig cells in future.

## Conclusions

Oxidative stress might be involved in TOCP-induced autophagy of mouse Leydig TM3 cells.

## Abbreviations

CYP, cytochrome P450; GSH, glutathione; GSH-PX, glutathione peroxidase; NAC, N-acetyl-L-cysteine; OPIDN, OP-induced delayed neuropathy; RIA, radioimmunoassay; ROS, reactive oxygen species; SCOTP, saligenin cyclic-o-tolyl phosphate; SOD, superoxide dismutase; TAS, total antioxidant status; TEM, transmission electron microscopy; TOCP, tri-ortho–cresyl phosphate; TOS, total oxidant status
